# Relationship of orthopedic examination, goniometric measurements, and radiographic signs of degenerative joint disease in cats

**DOI:** 10.1186/1746-6148-8-10

**Published:** 2012-01-27

**Authors:** B Duncan X Lascelles, Yaa-Hui Dong, Denis J Marcellin-Little, Andrea Thomson, Simon Wheeler, Maria Correa

**Affiliations:** 1 Comparative Pain Research Laboratory, College of Veterinary Medicine, North Carolina State University, Raleigh, NC, USA; 2 Center for Comparative Medicine and Translational Research, Department of Clinical Sciences, College of Veterinary Medicine, North Carolina State University, Raleigh, NC, USA; 3 Department of Population Health and Pathobiology, College of Veterinary Medicine, North Carolina State University, Raleigh, NC, USA; 4 College of Veterinary Medicine, North Carolina State University, Raleigh, NC, USA; 5 Graduate Institute of Epidemiology and Preventative Medicine, College of Public Health, National Taiwan University, Taipei, Taiwan

**Keywords:** Pain, Degenerative joint disease, Osteoarthritis, Feline, Goniometry, Orthopedic, Joint

## Abstract

**Background:**

Available information suggests a mismatch between radiographic and orthopedic examination findings in cats with DJD. However, the extent of the discrepancy between clinical and radiographic signs of OA in companion animals has not been described in detail. This study aimed to evaluate the relationship between orthopedic examination findings, joint goniometry, and radiographic signs of DJD in 100 cats, in a prospective observational design. Cat temperament, pain response to palpation, joint crepitus, effusion and thickening were graded. Radiographs of appendicular joints and the axial skeleton were made under sedation. Joint motion was measured by use of a plastic goniometer before and after sedation. Associations between radiographic degenerative joint disease (DJD) and examination findings were assessed to determine sensitivity, specificity and likelihood estimations.

**Results:**

Pain response to palpation was elicited in 0-67% of the joints with DJD, with a specificity ranging from 62-99%; crepitus was detected in 0-56% of the joints and its specificity varied between 87 and 99%; for effusion, values ranged between 6 and 38% (specificity, 82-100%), and thickening, 0-59% (specificity, 74-99%). Joints with DJD tended to have a decreased range of motion. The presence of pain increased the odds of having DJD in the elbow (right: 5.5; left: 4.5); the presence of pain in the lower back increased the odds of spinal DJD being present (2.97 for lumbar; 4.67 for lumbo-sacral).

**Conclusions:**

Radiographic DJD cannot be diagnosed with certainty using palpation or goniometry. However, negative findings tend to predict radiographically normal joints. Palpation and goniometry may be used as a tool to help to screen cats, mostly to rule out DJD.

## Background

Published information indicates that degenerative joint disease (DJD) is common in domesticated cats [1-15]. In humans, pain is the major clinical symptom in osteoarthritis (OA) and a key determinant for seeking medical care. This pain is the leading cause of mobility impairment in aging humans [16,17]. Relatively little is known about the direct relationship between DJD, pain and mobility impairment in companion animals. In a feline cruciate transection model of OA, ground reaction forces and limb kinematics recovered to pre-surgical levels over 1 year, despite progression of radiographic OA [18]. In a discussion of this model, it was indicated that after 5 years the joints have severe radiographic signs of OA without associated pain [19]. In contrast, several studies have identified cats with radiographic DJD and mobility impairment [2,9,20] and NSAID administration significantly improved mobility [2,9,20]. It appears that in some cats, radiographically apparent DJD is associated with pain and results in impaired mobility. However, available information suggests a mismatch between radiographic and orthopedic examination findings in cats with DJD. In a study by Clarke and Bennett, [2] 34% of joints assumed to be painful on manipulation during a orthopedic examination did not have any radiographic signs of osteoarthritis. In another recent study that evaluated 'radiographic DJD' and 'pain on manipulation', [9] 55 joints had radiographic signs of osteoarthritis (using radiographic features generally accepted for dogs), but only 18 of these (33%) were painful on manipulation. A similar discrepancy between radiographic features of OA and pain exists in humans, being best characterized for the knee [21-25] but also present in other joints [26].

The extent of the discrepancy between orthopedic examination findings and radiographic signs of OA in companion animals has not been described. Also, while we know how clinical signs predict radiographic signs of OA in humans, [25] this information is not known for companion animals.

The aim of this study was to evaluate the relationship between radiographic signs of DJD, orthopedic examination findings, and joint goniometry in cats.

## Results

Twenty-five cats in each age group were successfully recruited and included in the study. Of the 100 cats recruited, 18 were purebred, and 82 were domestic shorthaired or longhaired. The mean (± SD) age was 9.42 ± 5.07 years (range, 6 months to 20 years), and mean bodyweight was 5.13 ± 1.64 kg. (range, 2.08-10.16 kg). The median (range) body condition score (BCS) was 3 (1-5) out of 5. The temperament scores were 0 in 40% of the cats, 1 (18%), 2 (13%), 3 (24%), or 4 (5%). Due to this fact, incomplete pain scores were present in 8-15% of individual joints and spinal segments for a total of 207 missing pain scores out of 2000. Conscious goniometry could not be performed in 28 cats due to their temperament, but goniometry was performed in all cats when sedated. Radiographic assessment was complete in every cat.

The prevalence of radiographic DJD in this population has been described previously [10]. The number of affected joints, or spinal segments and associated manipulation scores for pain, crepitus, effusion and thickening (S_Pain_, S_C_, S_E _and S_T _respectively), as well as the number of joints with no abnormalities apparent on examination, are summarized in Table 1. The elbow and hip joints were most frequently found to be painful, followed by the stifle and tarsus. The lumbar and lumbo-sacral (L-S) segments were the most frequently painful segments in the axial skeleton. The elbow joint most frequently had an elevated S_C_, S_E_, and S_T_, followed by the stifle and tarsus. Cats with unfriendly temperament (scores 3-4 inclusive) had higher radiographic DJD (*P *= 0.005 and pain scores (*P *= 0.028) than cats with friendly temperament (scores 0-2 inclusive).

**Table 1:** Number of joints (percentage) with radiographic *DJD*, pain on manipulation, crepitus, effusion, or thickening among 100 cats.

Joints	Radiographic DJD (%)	Pain (%)	Crepitus (%)	Effusion (%)	Thickening (%)	Number of joints with no pain, crepitus, effusion or thickening
R Elbow	33 (33%)	36 (40%)	26 (26.8%)	19 (19.6%)	34 (35.1%)	50

R Shoulder	11 (11%)	10 (11.4%)	2 (2.1%)	NA	2 (2.1%)	89

R Carpus	17 (17%)	3 (3.3%)	4 (4.1%)	1 (1%)	4 (4.2%)	92

R Hip	69 (69%)	33 (35.9%)	5 (5.1%)	NA	NA	66

R Stifle	54 (54%)	19 (20.7%)	10 (10.1%)	13 (13.3%)	14 (14.4%)	74

R Tarsus	46 (46%)	16 (17.4%)	11 (11.1%)	13 (13.1%)	10 (10.1%)	78

L Elbow	36 (36%)	30 (34.9%)	18 (19.6%)	16 (17.4%)	31 (33.7%)	57

L Shoulder	17 (17%)	13 (15.5%)	4 (4.6%)	NA	3 (3.4%)	85

L Carpus	14 (14%)	5 (5.8%)	1 (1.1%)	3 (3.3%)	5 (5.4%)	91

L Hip	62 (62%)	32 (35.2%)	7 (7.2%)	NA	NA	65

L Stifle	48 (48%)	20 (22%)	14 (14.4%)	12 (12.4%)	16 (16.5%)	73

L Tarsus	39 (39%)	15 (16.5%)	9 (9.3%)	10 (10.3%)	8 (8.2%)	80

Cervical	20 (20%)	3 (3%)				96

Thoracic	43 (43%)	8 (8.7%)				87

Lumbar	26 (26%)	24 (26.4%)				42

L-S	29 (29%)	24 (26.1%)				68

*R*, Right; *L*, Left. *NA *= not assessed. Column showing number of joints with no pain, crepitus, effusion or thickening shows numbers out of 100; numbers in this column for axial skeleton are for pain only

There were significant associations between radiographic DJD and pain scores, which held true for the right and left elbows (P < 0.002) and the lumbar and L-S region (*P *< 0.032) when collapsed DJD_N/Y _and Pain_N/Y _were used in the analyses. There were significant associations between DJD_N/Y _and normal/abnormal S_C_, S_E_, S_T_, for both elbows (*P *< 0.002) and tarsi (*P *< 0.03) with the exception of the right tarsus S_C _(P = 0.054) and left elbow S_E _(P = 0.536). The SENS, SPEC, PPV, and NPV of normal/abnormal S_C_, S_E_, S_T_, values and Pain_N/Y _with regards to DJD_N/Y _are listed in Table 2. SENS was low overall, and was highest for Pain_N/Y_. PPV was highest for S_C _and S_T _but was generally low. SPEC and NPV were higher, suggesting that the absence of orthopedic examination findings of pain, crepitus, effusion and thickening could be used to rule out DJD with a high degree of certainty. The overlap of radiographic and orthopedic findings is listed in Table 3.

**Table 2:** Sensitivity, specificity, and predictive values (%) of orthopedic examination findings for the detection of DJD in 100 cats.

Joints	Pain				Crepitus				Effusion				Thickening			
	**SENS**	**SPEC**	**PPV**	**NPV**	**Se**	**Sp**	**PPV**	**NPV**	**Se**	**Sp**	**PPV**	**NPV**	**Se**	**Sp**	**PPV**	**NPV**

**R Elbow**	67	73	56	81	56	88	69	80	38	89	63	74	59	77	56	79

**R Shoulder**	22	90	20	91	10	99	50	90	NA	NA	NA	NA	0	98	0	89

**R Carpus**	0	96	0	84	6	96	25	84	6	100	100	84	20	99	75	87

**R Hip**	40	73	76	37	4	94	60	31	NA	NA	NA	NA	NA	NA	NA	NA

**R Stifle**	22	81	58	48	9	89	50	46	15	89	62	48	16	87	57	48

**R Tarsus**	22	86	56	58	18	94	73	58	24	96	85	60	20	98	90	60

**L Elbow**	56	78	60	75	38	91	72	72	21	84	44	64	47	74	52	70

**L Shoulder**	29	87	31	86	7	96	25	84	NA	NA	NA	NA	7	97	33	85

**L Carpus**	0	93	0	86	0	99	0	86	15	99	67	88	15	96	40	87

**L Hip**	33	62	56	39	10	97	86	41	NA	NA	NA	NA	NA	NA	NA	NA

**L Stifle**	19	75	40	51	15	86	50	53	7	82	25	49	15	82	44	52

**L Tarsus**	24	88	53	66	18	97	78	65	24	98	90	67	18	98	88	65

**Cervical**	12	99	67	82	NA	NA	NA	NA	NA	NA	NA	NA	NA	NA	NA	NA

**Thoracic**	14	95	63	6	NA	NA	NA	NA	NA	NA	NA	NA	NA	NA	NA	NA

**Lumbar**	43	79	42	81	NA	NA	NA	NA	NA	NA	NA	NA	NA	NA	NA	NA

**L-S**	50	82	50	82	NA	NA	NA	NA	NA	NA	NA	NA	NA	NA	NA	NA

*NA *= Not Assessed

**Table 3:** Numbers of joints and spinal segments with DJD on radiographs, pain on palpation, and with both among 100 cats

Joints	DJD only	Pain only	Both DJD and Pain
Shoulder	28	23	6

Elbow	69	66	38

Carpus	32	8	0

Hip	131	65	43

Stifle	102	40	18

Tarsus	85	31	17

Cervical	20	3	2

Thoracic	43	8	5

Lumbar	26	24	10

Lumbo-Sacral	29	24	12

ROM_C _and ROM_S _differed significantly for the stifle joints, right shoulder and right carpus, although the differences were small, ranging from 1° to 5° (Table 4). ROM_S _data were collected more rapidly than ROM_C _data (median, 17 min; range, 9-27 min vs. 25 min and 10-40 min, *P *< 0.0001).

**Table 4:** Mean (range) maximal range of motion (ROM) measured in cats when conscious (C-Max) and sedated (Sed-Max)

Joints	C-Max ROM	Sed-Max ROM	P value
R Shoulder	136 (93, 160)	137 (96, 158)	**0.041**

R Elbow	144, (74, 160)	143 (74, 156)	0.144

R carpus	184 (136, 210)	187 (115, 259)	**0.050**

R Hip	122 (88, 159)	123 (102, 147)	0.317

R Stifle	146 (110, 166)	149 (120, 167)	**0.001**

R Tarsus	147 (100, 163)	150 (123, 165)	0.140

L Shoulder	133 (112, 164)	134 (103, 154)	0.925

L Elbow	145 (96, 158)	143 (87, 153)	0.109

L Carpus	185 (155, 203)	187 (123, 208)	0.056

L Hip	120 (99, 150)	123.5 (98, 147)	0.183

L Stifle	144 (113, 165)	149 (118, 167)	**0.001**

L Tarsus	149 (130, 165)	149 (110, 168)	0.829

The odds ratios indicated that higher S_C_, S_E_, S_T_, and S_Pain _increased the likelihood of a joint having DJD present (Table 5). For example, cats with positive S_Pain _in the right elbow were 5.5 times more likely to have DJD compared to cats with negative S_Pain_. Similar increases were present for the left elbow, the lumbar, and lumbo-sacral areas. The likelihood of a joint having DJD was also increased for joints with positive S_C_, S_E_, and S_T_, particularly for the elbows and tarsi. Larger ROM_C _in shoulders, elbows and tarsi were associated with a lower likelihood of there being DJD present (*P *< 0.028) Larger ROM_S _in elbows, shoulders, carpi, and tarsi was associated with a lower likelihood of there being DJD present (P < 0.025).

**Table 5:** Influence of orthopedic examination findings on the likelihood (odds ratios) of there being radiographic DJD present in 100 cats.

Joints	Pain*	Crepitus*	Effusion*	Thickening*	C-Max ROM	Sed-Max ROM
R Shoulder					0.904	0.930

R Elbow	5.5	9.16	4.97 §	4.87	0.875	0.902

R carpus				19.75		0.918

R Tarsus			8.41	13.25	0.926§	0.936

L Shoulder					0.880	0.904

L Elbow	4.5	6.66		2.55 §	0.915	0.907

L Carpus			14.2 §			0.908

L Tarsus		6.44 §	18.0	13.1	0.886	0.792

Lumbar	2.97 §					

L-S	4.67 §					

Only statistically significant odds ratios are included

*Pain, effusion, crepitus and thickening scores were classified as present or absent

§ The significance of these odds ratios was lost due to 'age,' acting as a confounding variable

Age had a large effect (> 10% change in odds ratios) on the relationship of S_Pain_, S_C_, S_E_, and S_T_, and DJD_N/Y_. Weight, sex, temperament, and time point during the study had a minor effect (< 10% change in Odds ratios). BCS had a variable effect, but on average it was < 10% for all parameters, and it did not change the significance of the relationship between the parameter and DJD. For the appendicular skeleton, controlling for age decreased the likelihood that a joint with a positive orthopedic parameter (pain, crepitus, effusion, thickening) had radiographic DJD by between 28 and 37% (average changes: appendicular pain, -28%; crepitus, -29%; effusion -36%; thickening, -37%). This effect resulted in a change in significance (Table 5). Study time point (data collected in the first 4.5 months of the study compared to data collected in the second 4.5 months of the study) had the next largest effect on ORs for S_E _(11% increase on average) but not on other OR; however significance was not altered. For the axial skeleton, controlling for age decreased the likelihood that a positive pain response would be associated with radiographic DJD by between 3 and 76% (cervical, -76%; thoracic -3%; lumbar -42%; lumbo-sacral, -58%; average axial, -45%), and this resulted in a loss of significance for the lumbar and lumbo-sacral segments. When an interaction term was included in the model to evaluate OR, age was not shown to be an effect modifier, and stratification analysis supported this conclusion. Age had no confounding effect on the association between an abnormal ROM_C _or ROM_S _and the likelihood of a joint having DJD.

## Discussion

There is no validated assessment system to measure joint pain in cats although recently some progress has been reported in this [27]. In the present study, the level of aversive response interpreted as indicative of pain was evaluated subjectively during joint manipulation by a single investigator. The scores were recorded without consideration to viewing of the radiographs. This does not make it valid as an absolute measure, but the authors believe it is a valid comparison between different cats and different joints. There was no effect of time point of the study on the OR for the relationships between orthopedic findings and radiographic DJD, suggesting that the examining investigator did not change interpretation of parameters during the course of the study. Additionally, there did not appear to be any learned effect within each cat, and no biasing effect of sedation. Using this approach, it was found that the most frequently painful joints were the elbows and hips, and the most frequently painful spinal areas were the lumbar and lumbo-sacral segments. These data suggest that these would be the most impactful areas to target for further investigation as to the potential cause of pain.

Cats with higher S_DJD _and S_Pain _were less friendly than cats with lower S_DJD _and S_Pain_. Little work has been performed on determining how best to evaluate temperament in domesticated cats [28] and none on the association between temperament and disease. Several studies have evaluated aspects of behavior in relation to the presence of DJD [2,9,13,27] but none have evaluated temperament thus far. From the present study, one can speculate that higher pain scores are logically associated with a worse temperament, but the finding of a strong association between temperament and DJD_Y_/DJD_N _suggests that radiographic DJD is associated with pain, and thus unfriendly temperament. Having a valid measure of temperament, or change in temperament may be a useful surrogate measure of pain. Future work could evaluate this relationship further by looking at the effect of an analgesic on temperament in cats with DJD. Some work in humans has suggested increased aggression in association with OA [29] and treatment of OA with joint replacement has been shown to improve mood and well-being in human patients [30]. In the human field, there has been a recent interest in understanding the psychological effects of joint pain [31]. Our study is unique in its evaluation of veterinarian-assessed temperament, but limited and answers to the effect of chronic OA associated pain on temperament, mood, and aggression in companion animals deserve further exploration.

Significant relationships were found between pain on examination and radiographic DJD for the elbows, and the lumbar and lumbo-sacral areas. This may reflect a stronger association between disease and pain for these joints over others, or may reflect the fact that these joints may be easier to examine and manipulate appropriately. However, overall, the data indicated that the presence of signs of pain did not result in a high degree of certainty that radiographically evident DJD was present. This discordance has been suggested in other studies of cats with DJD, although the relationships were not described in detail [2,13]. This discordance between pain and radiographic signs is in line with studies in humans, although in human studies the pain is generally self-reported [21-25]. The discordance between pain and radiographic signs may be partly explained by the discordance between radiographic signs and grossly assessed signs of joint degeneration, as highlighted recently in cats [15]. The higher values for SPEC and NPV than SENS and PPV suggest the absence of pain (and crepitus, effusion or thickening) can be used clinically to help rule out DJD with a high(er) degree of certainty.

The presence of pain, crepitus, effusion or thickening was found to be associated with an increased the chance (ORs) of there being DJD present for certain joints. Age was found to be a confounding variable in the calculation of these ORs. As age is strongly associated with the presence of radiographic DJD, [10] it could be argued that controlling for age gives a more realistic overall view of the association between an orthopedic examination finding and the presence of radiographic DJD, avoiding the overestimation of the association between DJD and pain. It is clear from the human literature that as radiographic OA becomes more severe, there is a closer association between joint pain and radiographic OA, [24] and radiographic severity becomes greater with age. In a recent review of the association between knee pain and radiographic OA, [24] decreasing discordance with increasing age was seen in all the reviewed studies except one [32]. Given that we found no evidence of an effect modifier for age, the clinical interpretation should be that increasing age is associated with less discordance between radiographic and orthopedic examination findings.

Overall, the present study found that radiographic DJD is associated with decreased ROM in the shoulder, elbow, carpus and tarsus, and that there was no effect of age on these relationships. This suggests that this parameter holds true regardless of age. Although a previous study in clinically normal cats found no significant difference between conscious and sedated goniometric measurements in cats, [33] the present study found small differences. The magnitude of the differences between ROM measured in conscious and sedated cats would, from the results of this study, appear to be minimal, and likely have no clinical significance. However, further research would be needed to substantiate or refute this. The difference between the studies may be partly explained by the use of individual angles of flexion and extension for comparison in the former study, [33] and the use of ROM and the inclusion of joints with DJD in the present study. Increased ROM measurements were associated with decreased odds of there being radiographic DJD present, and therefore ROM may be of value in helping to rule out DJD.

## Conclusions

Overall, this study suggests that radiographic DJD cannot be diagnosed with certainty using palpation or goniometry. However, negative findings with respect to pain, crepitus, effusion and thickening, tend to predict radiographically normal joints. Increased ROM measurements were associated with decreased Odds of there being radiographic DJD present, and therefore ROM may be of value in helping to rule out DJD.

## Methods

The data presented here were collected during a study, evaluating the prevalence of radiographic DJD in domestic cats [10]. The study was a prospective, observational study using a database of 1640 cats from a single practice. Cats were divided into four age groups (6 months to 5 years; > 5-10 years; > 10-15 and > 15-20 years old). Within each age group, cats were assigned a number and then the cats in each group were randomly ranked. Then owners were contacted. The first 25 cats in each group whose owners were willing to participate in the study were included in the subset of 100 cats selected for this study. Owners were sent two recruitment letters at 1-month intervals and then contacted via telephone. If there was no response or they declined after initial contact, the next randomly selected owner was contacted and the sample completed with 25 cats in each age group. Once selected, owners visited our Veterinary Teaching Hospital (VTH) and each cat had a general physical examination. Age, weight, body condition score (BCS; out of 5) and sex were recorded. An orthopedic evaluation of the appendicular and axial skeleton was performed. The orthopedic evaluation consisted of careful palpation of every joint, with each cat being assessed by the same experienced assessor (BDXL). The same order was followed in every cat for the evaluation (right fore, right hind, left fore, left hind, axial skeleton). During the orthopedic evaluation, the pain response to palpation of every joint and each part of the axial skeleton was graded on the following numerical rating scale (pain scores, S_Pain_): 0 - no resentment; 1 - mild withdrawal; mildly resists; 2 - moderate withdrawal; body tenses; may orient to site; may vocalize/increase in vocalization; 3 - orients to site; forcible withdrawal from manipulation; may vocalize or hiss or bite; 4 - tries to escape/prevent manipulation; bite/hiss; marked guarding of area. Additionally, each appendicular joint was evaluated for crepitus, effusion and thickening on a scale of 0 - none; 1 - slight - moderate; 2 - severe, generating crepitus scores (S_C_), effusion scores (S_E_) and thickening scores (S_T_). Collectively, these scores were termed 'manipulation scores'. Additionally, a temperament score was given to each cat as previously described, [33] where 0 = neutral attitude, purring, kneading; 1 = resistance to restraint; 2 = resistance to restraint, growling and hissing; 3 = resistance with biting and scratching, hissing, spitting, and vocalizing; and 4 = resistance with biting, scratching, vocalizing, spitting, hissing urinating, or defecating. Examination (and goniometry) was performed with the cat in lateral recumbency, using minimal restraint provided by a single assistant.

Following the physical and orthopedic examination, goniometric measurement of every appendicular joint (excluding joints within the manus or pes) was performed using a plastic goniometer with 1-degree increments (Baseline 360° clear plastic 6 in. goniometer, AllegroMedical, Mesa, AZ). Goniometry was performed as previously described [33] in conscious cats and measurements were made of maximal angles of flexion and extension. Only the principal investigator performed all the goniometry assisted by one assistant (AT) who provided only the amount of restraint necessary. The time necessary to perform goniometry was recorded.

Following the physical examination, each cat was sedated for radiographic examination using a combination of ketamine (3-5 mg/kg), butorphanol (0.3-0.4 mg/kg) and medetomidine (10-15 mcg/kg) administered intramuscularly. Orthogonal radiographs of all joints and the spine were made under sedation using indirect digital flat panel imaging system (Canon Medical CXDI-50 G Sensor, Eklin Medical Systems, Santa Clara, CA). Criteria for evaluation of radiographic signs of feline appendicular joints and axial skeleton DJD were as previously described, [10] and based on the results of other studies that have evaluated the relationship between radiographic DJD and aspects of joint destruction [14,15]. Radiological features that were considered indicative of the presence of DJD in appendicular joints were: joint effusion, osteophytes, enthesophytes, joint-associated mineralization, sclerosis, subchondral bone erosions or cysts and presence of intraarticular mineralization. Radiological features indicative of axial skeleton DJD were osteophytes, spondylosis, disc-associated degeneration (end plate sclerosis, erosion, disc mineralization or narrowing) and subluxation. Using these criteria, each joint and part of the axial skeleton of each cat was evaluated independently by two board-certified radiologists and a board-certified surgeon. Appendicular joints evaluated were carpus, elbow, shoulder, tarsus, stifle and hip. The axial skeleton was evaluated by dividing the spine into cervical (C1-C7), thoracic (T1-T13), and lumbar (L1-L7) segments and lumbo-sacral area (L7-S1). A subjective radiographic DJD score (termed 'overall DJD score') from 0-10 (0 - No radiographic abnormalities identified; 10 - ankylosis) was assigned to each joint and each part of the axial skeleton based on the presence of radiographic changes and their severity [10]. The median values for the DJD scores obtained from the 3 observers' assessments were used in statistical analyses. Agreement between the observers has been discussed previously [10]. The goniometric measurements of all joints were repeated after radiography while the cats were still sedated. The time necessary to perform these measurements was recorded. Data were collected over a 9-month period of time.

## Statistical analyses

Non-parametric statistical tests were used as the data consisted of mainly categorical variables. Categorical variables were collapsed if data were sparse. For example, the relationship between DJD and both pain scores and temperament was evaluated by collapsing the temperament scores to 'friendly' (scores 0-2 inclusive) and 'unfriendly' (scores 3 and 4); Wilcoxon rank sums tests were used then to determine the association of DJD and pain scores. The overall DJD scores (0-10) assigned for each appendicular joint or spinal segment were re-grouped on a 1-5 scale as follows: 0 (1, none); 1 (2, trivial); 2-4 (3, mild); 5-7 (4, moderate); 8-10 (5, severe). The DJD data were also defined as 'no DJD' (DJD_N_) and 'DJD present' (DJD_Y_). The pain scores (0-4) were regrouped into 'no pain' (Pain_N_) or 'pain present' (Pain_Y_). The other manipulation scores (0-2) were regrouped into 0-1: 0 - no abnormal signs; 1 - abnormal signs present. Goniometric measurements in the conscious and sedated states were used to calculate range of motion (ROM), producing conscious maximal ROM (ROM_C_) and sedated maximal ROM (ROM_S_) values for each appendicular joint. Chi-square test or Fisher's exact tests were used to identify associations between DJD scores and manipulation scores (S_P_, S_C_, S_E_, S_T_). Using DJD_N_, DJD_Y_, S_P_, S_C_, S_E_, and S_T _scores regrouped into binary variables (yes/no), sensitivity (SENS), specificity (SPEC), positive predictive value (PPV), and negative predictive value (NPV) for determining if DJD was present were calculated accordingly. Wilcoxon rank sums tests were used to compare ROM_C _and ROM_S _and to compare the time it took to collect ROM_C _and ROM_S _measurements. Logistic regression was used to determine the likelihood of DJD occurrence expressed as odds ratios and S_P_, S_C_, S_E_, S_T _and ROM. Sex, age, weight, BCS, temperament, and time point of the study (in an attempt at controlling a learner's effect due to the orthopedic examination performed in a sequence and therefore to check for the effect of early or late time points in the study) were added to the regression models as potential confounders. Confounding effect was set as a 10% change of the odds ratio by the investigators, a priori. Analyses were performed using SAS (SAS version 9.1, SAS Institute Inc., Cary, NC). An alpha value of ≤ 0.05 was set for statistical significance in all analyses.

## Abbreviations

DJD: Degenerative joint disease; OA: Osteoarthritis; BCS: Body condition score; S_Pain_: Pain score; S_C_: Crepitus score; S_E_: Effusion score; S_T_: Thickening score; DJD_N_: DJD not present in a joint; DJD_Y_: DJD present in a joint; Pain_N_: Pain not present in a joint; Pain_Y_: Pain present in a joint; ROM: Range of motion; ROM_C_: Maximal range of motion measured in the conscious cat;ROM_S_: Maximal range of motion measured in the sedated cat; SENS: Sensitivity; SPEC: Specificity; PPV: Positive predictive value; NPV: Negative predictive value.

## Competing interests

None of the authors believe their interpretation or presentation of the data was in any way influenced by any financial competing interests. Dr Simon Wheeler is an employee of Novartis. Drs Lascelles and Marcellin-Little have received honoraria in connection with sponsored Continuing Education seminars. Dr Lascelles received funding for this study from Novartis Animal Health, and has received other research funding from Novartis Animal Health. Dr Lascelles has acted as a consultant for Novartis Animal Health.

## Authors' contributions

BDXL and SW conceived and designed the study; BDXL and AT collected the data; GD and MC performed the statistical analysis; DJM-L assisted in data collection methods and helped draft the manuscript; all authors read, contributed to and approved the final manuscript.
